# Message from SNM President

**DOI:** 10.4103/0972-3919.63589

**Published:** 2010

**Authors:** BR Mittal

**Affiliations:** Department of Nuclear Medicine and PET, PGIMER, Chandigarh President, Society of Nuclear Medicine (India) Email: brmittal@yahoo.com

Dear Friends,

The Society of Nuclear Medicine (India) [SNM (I)] has entered the forty-second year of its existence since its inception in 1968. It is a fairly young and small society with its membership of 800 professionals incorporating scientists, physicists, technologists, biologists, pharmacists, and physicians. Inspite of the young age and small society, we have many achievements to be proud of; the most significant achievement being, the recognition of nuclear medicine as a broad clinical specialty by the Medical Council of India and National Board. We are self-reliant in human resource development in nuclear medicine. Many countries in the world still do not have formal physician and technologist training programs. We have post-doctoral research facilities (PhD), postgraduate (MD / DNB / DRM) residency programs, and technology courses (MSc / DMRIT / DNMT / BSc) in the specialty. We have four active zonal chapters and organize a national conference every year in different parts of the country. This year the forty-second annual conference of SNM (I) will be held in Chandigarh 'The City Beautiful'. If you ask me, the most important benefit of an SNM (I) membership is in fact the free subscription of the Indian Journal of Nuclear Medicine (IJNM). IJNM has witnessed the steady scientific growth of nuclear medicine in India. For many years, IJNM has been a mirror of the scientific culture of our society.

The Indian Journal of Nuclear Medicine is the right of every SNM member. As president, I assure you of the regular publication of IJNM, and getting it indexed is our top priority. I am happy that a new editorial team is committed to the cause and is bringing the current issue of IJNM on time. I take this opportunity to request you to kindly contribute articles based on your observations, rich experience, and rare encounters. I congratulate and appreciate the editorial team for their efforts.

Wishing you happy reading!

